# Providers' approaches to contraceptive provision in Cape Town

**DOI:** 10.3389/fgwh.2022.917881

**Published:** 2022-09-14

**Authors:** Kulthum Fataar, Virginia Zweigenthal, Jane Harries

**Affiliations:** Department of Public Health and Family Medicine, Faculty of Health Sciences, University of Cape Town, Cape Town, South Africa

**Keywords:** family planning, contraceptive provision, contraceptive counseling, health care providers, South Africa, qualitative research

## Abstract

**Background:**

Health care providers can play a significant role in empowering women to make informed decisions when selecting suitable contraceptive methods during contraceptive counseling. This study explores primary care providers' perspectives and approaches to contraceptive service provision for women attending public sector clinics in South Africa, with the intention of ascertaining established practices and training needs.

**Methods:**

Ten in-depth interviews were conducted at five primary health care facilities in urban areas in Cape Town, South Africa. Eligible participants included nurses providing contraceptive services and willing to participate in the study. The qualitative software package NVivo was used to sort and manage data. Data was analyzed using a thematic analysis approach.

**Results:**

Overall, providers emphasized supporting women in contraceptive decision-making. Sexual and reproductive health training increased providers confidence to deliver appropriate contraceptive services. Contraceptive prescribing practices were influenced by women's medical history and preferred bleeding patterns. Providers' concerns about adherence to methods for younger women and suspected adverse events for older women impacted on prescribing. Challenges experienced when providing contraceptive services included: contraceptive stockouts; time constraints of employed women accessing the service; and their work pressure due to providing other health services.

**Discussion:**

Health care providers play a critical role in facilitating women's right to access high quality contraceptive services. Providers saw themselves as negotiators during contraceptive counseling. They considered both women's preferences and their own recommendations for contraception, to provide information that would enable women to make informed contraceptive decisions. By reinforcing this approach to contraceptive counseling and focusing on shared decision-making, should encourage autonomy in method selection and limit the influence of provider's contraceptive method selection.

## Introduction

Universal access to family planning services is critical to meet the sexual and reproductive health needs and desires of reproductive aged women. In 2019, globally, 190 million women of reproductive age wanted to avoid unintended pregnancy but were not using a contraceptive method (1). Reported reasons for non-use include misperceptions about the risk of pregnancy, concerns related to contraceptive side effects, partner resistance and, conflict between religious beliefs and use of contraception (2). Increasing contraceptive uptake could prevent millions of unintended pregnancies (2), minimize the risk of maternal and infant mortality (3, 4), and prevent adverse health outcomes from accessing unsafe abortion services in contexts where abortion services are unavailable or difficult to access (5).

In South Africa (SA), a 2016 household survey found the modern contraception-use prevalence rate was 47.9% amongst all women of reproductive age (15–49), which when compared to the 2003 rate of 50.1%, indicates a decline of 2.2% (6, 7). In SA, modern contraceptive methods are available at no cost in the public sector health services and include male and female condoms, injectable progestogen contraceptives, intrauterine devices (IUD), sub-dermal contraceptive implants (implant), oral contraceptives (OC) and sterilization (8). Notably, not all the listed contraceptive methods are offered at all public health facilities and methods may not always be available due to stockouts and lack of provider training in some methods (7). Amongst women using modern contraception, 16% of all women were using the depot medroxyprogesterone acetate (DMPA) injectable in 2016. Thus, DMPA remains the most used modern contraceptive method (6). In recent years, there has been significant debate about DMPA's role in increasing the risk of HIV acquisition amongst women using this injectable method (9–11). However, in 2019, the World Health Organization (WHO) released a statement advising there is no increased risk of HIV acquisition amongst DMPA users (12). Increasing access to accurate and comprehensive reproductive health information is important to empower women to make informed decisions about contraceptive method choices.

According to recent findings in SA, 12% of reproductive age women desired to limit or space their pregnancies but were not using contraception (6). Consequently, many women continue to experience unintended pregnancies (6, 13, 14). In 2019, the National Department of Health (NDoH) updated the National Contraception Clinical Guidelines (8). The updated policy, building on the 2012 guidelines, which expanded the availability of contraceptive methods (including longer-acting methods) in the public health sector such as the implant, introduced in public health facilities in 2014 (15), includes support for users' agency and aligns local medical eligibility criteria (MEC) with the 2015 World Health Organization (WHO) MEC (8). The WHO MEC promotes the use of hormonal injectables in any circumstance; and, the use of progestogen oral pills (POC) or implants for breastfeeding women (16).

Increased risk of unintended pregnancy is not limited to non-users of contraception; inconsistent and incorrect contraceptive use may also result in unintended pregnancy (7). Inconsistent use may result from inadequate reproductive health information provided to women during contraceptive counseling. Previous South African studies found that many women report receiving insufficient information about their chosen method when accessing family planning services (7, 17, 18). Furthermore, women also discontinued contraceptive use due to unwanted side effects (6, 7, 17), and many desired more information about side effects and their management from health care providers (providers) (17). Furthermore, amongst women attending public sector clinics, only 56.4% of women were provided with information about side effects and 47.9% were advised how to manage these (6). These findings are alarming given that providers delivering contraceptive services within clinics play a significant role in facilitating the process of contraceptive decision-making. Their work includes providing information about the various contraceptive methods available, the possible side effects associated with each method and its suitability for women.

As most reproductive aged women in South Africa access family planning services from the public health sector, providers' recommendations are powerful and could contribute to women selecting a contraceptive method (19). Consequently, the attitudes providers have toward contraceptive methods may influence or restrict women's contraceptive choices. These biases may not be evidence-based or have medical reasoning (20).

For example, one study found that providers are more likely to recommend a two-month injectable to younger women citing return to fertility is faster in comparison to the three-month injectable despite recommendations that women should not be limited to use either injectable based on age (8, 18). Providers also restricted access to contraception if women were not menstruating, as lack of menstruation was used as a measure to determine possible pregnancy (18). Other possible biases include providers questioning the suitability of OC for younger women, as they are “forgetful” (21) or providers' personal experiences with a specific contraceptive method may influence their recommendations (21). Furthermore, providers' lack of adequate knowledge and training, level of confidence and access to updated clinical guidance may also influence their recommendations and delivery of sexual and reproductive health services (20, 22). For example, providers who have been trained to perform IUD insertion may be more likely to recommend this method (22). Lastly, external factors may influence providers recommendations including sociocultural norms and health system challenges (20).

To gain a deeper understanding of how providers influence delivery of contraceptive services, this study explores primary care providers' perspectives and approaches to contraceptive service provision for women attending public sector clinics in SA. Ascertaining their perspectives and experiences could explain their prescribing practices and inform interventions and training to enhance good practice.

## Methods

### Study design, research setting and study population

We conducted a qualitative study with primary health care providers (providers) at five nurse-based public sector health care facilities, delivering free contraceptive services, in addition to a range of personal health services, spread throughout metropolitan Cape Town, SA. Prior to the recruitment process, we scheduled a meeting with health managers who provided a list of health care facilities providing contraceptive services and key personnel to contact to assist with study recruitment. We obtained approval from area managers to contact facility managers within selected areas. Most facility managers preferred to inform eligible providers about the research study themselves and negotiated an appropriate day and time for an interview if providers indicated their interest. Eligibility criteria included providers delivering contraceptive services and willingness to participate in the study. Each sampled clinic had two providers delivering contraceptive services; thus, only two providers were recruited from the five selected facilities. All ten providers recruited agreed to participate in the study.

We used purposive sampling to approach staff working in a range of facilities based on geographical location and the diverse patient population accessing contraceptive services including youth, migrants, and women utilizing clinics proximal to their workplace. Facilities were selected to determine how the diversity of patient characteristics and their contraceptive needs influenced the perceptions and experiences of providers.

### Data collection

A semi-structured interview guide was developed by the first author (KF) and reviewed by the third author (JH). Additionally, the guide was piloted with two providers who were later included in the study sample and re-ordered for flow and clarity. Prior to the interviews, providers were asked to share their sociodemographic information including their professional background and years of experience. During the interviews, they were asked about their training, how they conducted contraceptive counseling sessions and what factors they considered when making contraceptive prescribing decisions.

A total of ten individual in-depth interviews were conducted by KF, a female, English-speaking, Master of Public Health student trained in qualitative research methods. Interviews were conducted between October and November 2019. Due to English being a common language used in healthcare settings in SA, all providers agreed to the interview being conducted in English. Interviews were conducted in a private room at the facility and varied in duration between an hour and 90 min. Providers working at the same facility were interviewed on two separate days to avoid disrupting services.

Two vignettes were developed based on existing literature related to health care providers who are involved in family planning services in an African context (22). They were presented to providers prior to exploring their prescribing practices at the appropriate moment of the interview. The interview guide and vignettes are found in Supplementary material.

Vignettes present fictional characters as cases to explore the underlying knowledge, beliefs and attitudes of participants which might be challenging to identify when reporting on their personal experiences (23). They are useful when exploring how health professionals make clinical judgements or to identify biases toward patients or treatment (22, 24) and have been used to explore provider bias in contraceptive provision elsewhere (22). To limit socially desirable responses, vignettes should focus on asking providers how they would approach a specific clinical situation and that the “client” description has various characteristics beyond the specific characteristic (for example, age) the researcher is attempting to assess to avoid providers identifying the focus of the vignette (24).

In this study, the vignettes provided opportunity for further exploration into providers underlying attitudes and what factors they considered when recommending a specific contraceptive method. The first vignette aimed to explore how providers would approach counseling a female adolescent requesting OC, as it has been widely cited providers often question the suitability of OC for younger women (20, 25, 26). In view of research that reported that providers are more likely to recommend the IUD for women who have previously given birth (27, 28), the second vignette aimed to explore how providers would approach counseling a young woman who is primiparous requesting the IUD. Following the presentation of the vignettes, providers were asked to explain why they agreed or disagreed with the requested contraceptive method and if they disagreed, what alternative method would they recommend.

### Ethical considerations

We obtained ethical approval for the study from the Human Research Ethics Committee at the University of Cape Town (HREC REF 536/2019). Permission was also obtained from the health authority in order to access facilities. Prior to the interviews, written informed consent was provided by all study participants including permission for audio recording. Providers were also assured that to maintain confidentiality, identifying information would not be included in any research report, including their names, the facilities at which they work. We stored signed informed consent forms in locked cabinets. Digital data, including recordings and transcriptions, were stored in password protected files on a private, password-secured computer. Access to digital data was limited to the research team and a professional transcriber. We deleted all audio recordings after transcribing was completed and verified.

### Data analysis

The audio recordings of the individual interviews were transcribed by an independent transcriber. The first author listened to all audio recordings and reviewed all transcripts for accuracy and quality. Reflexive memoing was conducted by KF throughout the data collection and analysis process. All sorting, management and coding of data used NVivo (QSR International) qualitative software. We developed a codebook based on *a priori* codes derived from questions in the interview guide. The codebook was further refined after inductive codes were derived from the data. We used a thematic analysis approach was to identify and examine key themes and sub-themes emerging from the data. Illustrative quotes are provided for each theme. Informants are identified by their rank and years of experience, with PN for professional nurse and SN for staff nurse. PN's have four years training and work autonomously in specific areas including contraceptive prescription. SN's have 2 years training and work under the supervision of a PN.

## Results

### Facility and participant characteristics

Amongst the five facilities selected, three facilities provided all the contraceptive methods available in public-sector facilities including progestogen-only injectables, OC including combined oral contraceptive pills (COC) and POC, male condoms, intrauterine contraceptive devices (IUD) and the implant. Two facilities provided all the contraceptive methods excluding the IUD; one facility provided injectables and OC only.

Ten providers were interviewed, two from each facility. All were female and included one registered staff nurse (SN) and nine registered professional nurses (PN's) who provided contraceptive services. The median nursing work experience amongst providers was 8 years. In line with the services offered at their facility, in addition to family planning services, providers delivered a range of services including child health, women's health services such as antenatal and postnatal care, youth-centered services, cervical smears and breast examinations and diagnosis, treatment and management of HIV and tuberculosis and chronic health conditions.

The key themes identified during data analysis included the influence of contraceptive knowledge on providers practice, attitudes toward providing contraceptive services and factors which influenced prescribing practices. Sub-themes are explored within each key theme.

### Influence of contraceptive knowledge on practice

Providers emphasized that contraceptive knowledge prepared them to confidently deliver comprehensive contraceptive services. This knowledge came from prior sexual and reproductive health (SRH) training and clinical guidelines.

#### Sexual and reproductive health training

All providers had prior knowledge of contraception acquired through their studies, work experience and/or training. When commencing this work, they relied on previous knowledge, which gave them confidence to deliver an effective service. Over the course of their employment, providers who delivered contraceptive services were expected to complete an additional six-month SRH training course. After this training, many reported they found their prior knowledge did not sufficiently prepare them to provide contraceptive services that promoted women's contraceptive preferences:

…* except for you know how to inject … you don't really have that background knowledge of choosing what is best for the client. Whereas when you get this [SRH training]course you have a better understanding of how to, to treat*. (PN, 3 years)

Furthermore, many providers reported the training challenged common misconceptions about the suitability of certain contraceptive methods for women. Consequently, the training prompted reflection on prescribing practices and to critically reassess if these aligned with women's contraceptive needs and desires. For example, one PN who attended the training reported that clinic staff deterred providers from prescribing implants for younger first-time contraceptive users which differed from the training information received:

… *[Clinic staff] would say ‘never put in an [implant] for a 16 year old that's never had family planning'. And when I did sexual reproductive training [SRH trainers] said like why not? …. So, there's some myths that … was clarified … I sat in that training, I was like – now why do people say that, like why? Why are [nursing staff], like so resistant to do certain things and we can*. (PN, 4 years)

Additionally, one PN with 3 years' experience, who recently enrolled in the training emphasized that providers delivering contraceptive services needed additional SRH training“*… because [clinic staff] are mis-prescribing, we are giving wrong information, we're not giving enough information with regards to reproductive health*”, despite their previous knowledge gained from their professional qualification and training. This indicates that some relied on training to ensure their contraceptive knowledge was up to date, and most providers who attended SRH training were more confident to prescribe suitable contraceptive methods for women. This included the five providers who received training on implant and IUD insertion and removal. Those who had undergone IUD training often promoted the IUD to first time contraceptive users as well as those considering switching to a non-hormonal contraceptive method due to side effects. Conversely, providers who had not received training felt less confident to promote the IUD to women despite having theoretical knowledge of the IUD, and would only provide information and not prescribe the IUD when requested:

…* but personally me, I won't [prescribe the IUD]. The IUD, I'm not supposed to do that, but I don't really advertise as much you know, but at the end of the day it's still, if they ask for the information I will give them. But I won't … I don't like to do things that I'm not able to stand my ground*. (PN, 11 years)

#### Clinical guidelines

In addition to their knowledge acquired through training, most providers also used algorithmic clinical guidelines to assist with clinical decision-making. Some reported the guidelines increased their confidence in clinical decision-making. However, others reported that the guidelines were only suitable for the “*normal*/*ideal patient”* and were not tailor-made for all women. Furthermore, some associated the use of clinical guidelines with being “*computerized” –* to follow the decision-tree based assessment as opposed to exploring women's specific contraceptive needs:

…* Each patient is different and … the PACK [clinical guideline] isn't always like about the patient … you can follow the PACK but there's some cases where it's not like as in the algorithm … like I think that [providers] have been computerized … I think that we've driven away from what the patient needs and each patient is different. And we try and generalize and that's the biggest mistake*. (PN, 4 years)

Consequently, at times, some providers overlooked clinical guidelines in favor of prioritizing women's personal contraceptive needs. For example, one PN with 33 years' experience, questioned why women older than 40 years should switch from injectables to POC because it is “*…stated in a book somewhere”* and reported that she would prioritize women's preferences “*if [the patient] wanted to stay on it because [they] feel safe, that's fine with me, I will give it”*. Conversely, others reported that women older than 40 years should be counseled to switch from injectables to POC, basing this on “clinical guidelines” that suggest that injectables might result in reduced bone density. However, the risk of osteoporosis and risk of fracture remains uncertain in long-term users of injectables, such as depot-medroxyprogesterone (29).

Providers' contrasting opinions highlights how they weighed clinical knowledge and judgment against women's contraceptive needs. One PN maintained that she would not provide COC to women who have hypertension regardless of their choice as she needed to use clinical judgment to prescribe methods for women who could have adverse health outcomes:

*But if [patients] choose oral contraceptive while there are things that are hindering me to prescribe this oral contraceptive, I won't provide it. because at the end of the day, let's say they have a raised blood pressure that is part of the side effects of the oral contraceptive. So for me to … put your life at risk as a patient, I wouldn't be able to sleep at night knowing that I killed a patient*. (PN, 7 years)

Providers were generally not flexible about recommending COC's if it was contraindicated after assessing a woman's clinical history as they perceived the risk of an adverse event to be high and possibly dangerous. An alternative, more suitable contraceptive method then would be recommended. In comparison, providers had varying levels of flexibility for women over 40 years old continuing to use injectables, with some feeling more comfortable to continue prescribing injectables despite “clinical guidelines”. Providers also described their dilemmas when, based on medical history, women's preferred contraceptive methods were not suitable for them, yet women would insist on using it. In these situations, providers attempted to educate women about contraindications or provided them with booklets to read at home. However, if women refused alternative methods, they would document this and prescribe the preferred method as they reported “*at the end of the day, it's the patient's choice”*. Thus, despite many providers emphasizing that women have the right to choose their desired contraceptive method, they also used their clinical knowledge and experience to counsel women to use a more appropriate method, particularly when contraindications were present.

### Attitudes toward providing contraceptive services

Most providers enjoyed engaging with women about their sexual and reproductive health, providing health education and supporting women in their reproductive health choices. They maintained that access to contraceptive services was essential for women, including younger women, as it would allow them to space childbearing and avoid the socioeconomic impact that unintended pregnancy could have on both mother and child:

*I think it's an absolute necessity … it doesn't matter what age you are, people need to be able to have the right to decide when they want a child … or if they want children at all. I think it is part of, one of the building blocks in our communities. Because if we are not going to allow any lady to not be able to decide when she wants, she's going to end up with children that she's not going to be able to care for. She's not going to be able to care for herself*. (PN, 33 years)

Consequently, many providers were supportive of family planning decisions being centered on women's reproductive needs and desires through exploring their contraceptive preferences. They perceived themselves as negotiators who assisted women to make contraceptive decisions by delivering accurate reproductive health education that accounted for both women's preferences and provider's recommendations:

*You negotiate yes, but it must come from them, you need to know what she wants. I can't say this is good for you because of this benefit – but it's not what [the patient] came for, [they] wanted the 2 months [injectable] but you're advising for 3 months [injectable] … so let them talk, let's just share*. (PN, 7 years)

Thus, many providers disagreed with “*forcing*” women to use a contraceptive method they had not requested as they believed this would result in non-adherence. Consequently, to improve adherence, they felt that they should prescribe contraceptive methods tailored to women's choices:

…* But what I have seen is you cannot force a woman, if she does not want to use it,. Because if you give her the tablets and she doesn't want to take it, she's not going to take it. So, don't force yourself, give the patient's what they want so they can be compliant on it*. (PN, 5 years)

#### Contraception provision to younger and older women

Despite providers reporting the importance of negotiating with women about their desired contraceptive method, they displayed varying attitudes toward the suitability of contraceptive methods for specific age groups. Responses to the vignette of a 17-year-old female adolescent requesting to use COC demonstrated positive attitudes toward prescribing COC to younger women, but also highlighted perceptions of younger women's inability to be “responsible”. Many believed COC should be prescribed for “*mature*”, “*responsible”* and “*organized”* women who “*have a routine”* whereas younger women were described as “*forgetful”* and lacking a structured, daily routine. Consequently, providers probed younger women's adherence to other medication regimens to assess their ability to use COC consistently. Most would counsel younger women to use injectables instead, and some also suggested longer acting methods such as the implant and IUD. However, many also stated they would prescribe COC if women refused any other method as their main concern was the risk of an unintended pregnancy. Generally, providers advocated for and encouraged younger women's access to contraceptive services by establishing an effective youth-centered family planning service to avoid teenage pregnancies. Nonetheless, providers preconceived notions of younger women influenced their contraceptive counseling approach and prescribing practices, which might not be in young women's best interest.

Similarly, providers attitudes toward older women also influenced their contraceptive counseling approach and prescribing practices. Many reported that women over the age of 40 years old preferred injectables and refused to switch to POC, which was perceived to be a safer contraceptive method for older women. As reported above, while some providers were flexible about continuing injectables, others became frustrated with women who were reluctant to switch to POC, despite receiving education about the perceived possible health risks (such as reduced bone density) associated with the injectable. This resulted in these providers questioning their approach to contraceptive counseling as they felt they failed to persuade older women to switch to a perceived safer method:

…* Most [older women] have already came in with the mind set of I am not changing, and I think that is the, the problem that we have. We have so many older women in their 40's, 50's, not wanting to change from [injectable progestogen] to [progestogen only OC] which is a safer method for their age ... they say no, no sister I will maybe consider it at the next time but today, give me my family planning. So, they're very adamant like I don't know, if our approach is wrong or I really don't know*. (PN, 4 years)

In summary, despite providers voicing they promoted women's empowerment to choose their preferred method, options were limited due to their assessment of contraindications to specific methods. Consequently, women's contraceptive preferences were not always be prioritized, and method choices needed negotiation.

### “Every woman is different”: Factors influencing prescribing practices

Providers identified specific factors they considered when prescribing suitable contraceptive methods. These were women's clinical history, minimizing unwanted side effects and women's preferred bleeding patterns.

#### Clinical history

Providers identified women's clinical history as being the main factor they prioritized when prescribing contraception. This included asking women about their chronic illness history, current medication use and HIV status. One PN with 23 years' experience avoided prescribing OC for women using multiple chronic medications because “*adding another pill”* added to their oral medication burden. A few avoided the implant for HIV-infected women due to contraindications with one commonly prescribed antiretroviral drug at the time, efavirenz, which reduced the effectiveness of the implant, and rather prescribed injectables for these women. They also removed implants from HIV-infected women. Many found the changing guidelines that recommend adding condoms for HIV-infected women using implants, confusing and unrealistic, as they maintained that HIV-infected women were not compliant with condoms. Consequently, as previously highlighted, providers perform a balancing act by drawing on clinical guidelines, professional experience and their own clinical judgment for each individual woman's specific circumstances.

#### Minimizing side-effects

Many highlighted that women often requested specific methods based on recommendations by female friends and family members. They noted that women struggled to understand the differing side effects experienced by women using the same method. They emphasized that “*every woman is different*” and that it was challenging to predict the physical effects of a prescribed hormonal contraceptive method:

*But for family planning, there's hormones involved and not everybody's hormone levels are the same and not everybody's mood swings are the same – you may have moods, I don't have moods; you may have heavy bleeding – I don't have heavy bleeding. Even though we are both on contraception..*. (PN, 23 years)

Consequently, many strongly emphasized the need to provide health education, particularly focusing on possible side effects of methods. Education on side effects was important as providers considered this was the main reason women discontinued contraception:

*Because if you are not given the proper information when you start … we have a lot of people here who start on the injection and then they just don't come again and then you find out they started bleeding … some people get a period, others don't. Some people gain weight, others stay the same. Some people have, most people have irregular periods for the first 4 to 6 months, but we can treat that until your body is accustomed to it”*. (PN, 33 years)

#### Bleeding pattern preferences

Across all interviews, irregular and unwanted bleeding was seen as the main side effect for method discontinuation. Consequently, providers often asked women about their preferred bleeding pattern, as many requested specific contraceptive methods based on preferences, such as no bleeding or bleeding patterns that were “*regular*”, mimicking a regular menstrual period cycle. For example, one PN thought the primary motivation for many women requesting injectables was to avoid bleeding, and family planning was secondary to this:

*And patients who don't want a period … That's why most of them are on it, not because they are sexually active – because of her period … they are the ones [that say] ‘Sister, I don't want a period” because that's what they tell me. But it's a family planning method. ‘Yeah sister, and that as well, but I don't want a period'*. (PN, 5 years)

Another important factor reported was access to sanitary products, which were usually purchased by the parents of younger women. Providers reported that women were often concerned that reduced or excessive bleeding, resulting in use of sanitary products less or more frequently, may cause their parents to become suspicious that they were using contraception. Furthermore, one provider highlighted a woman who preferred no bleeding considered the injectable to be more economical because it was “*free*” and sanitary pads were not affordable.

### Health system challenges' influence on contraceptive delivery practices

Many providers experienced health systems related challenges which affected their ability to provide comprehensive contraceptive services. This included contraceptive stock outs and limited counseling time.

#### Contraceptive stock-outs

Despite providers wanting to provide women with information on all the contraceptive methods available and their side effects, as well as exploring women's preferences and needs, they felt this was mostly not possible. They highlighted challenges that impacted on service provision and might compromise women's contraceptive compliance. For example, contraceptive stockouts were repeatedly reported as a significant challenge, which limited women's ability to choose their preferred method. This resulted in forcing women using a method they did not choose and resistance to the alternatives. Occasionally, stockouts resulted in some women not returning for their follow-up appointment:

*Yes and there was a time that ... the two-month injection was out of stock for maybe 2 to 3 months, then the client must be forced to take something else and some of them stayed away. There was also a time that the [oral contraception] was out of stock now for 3 months. So, it was also not nice because now the lady must go onto the injectable and some of them decided, no they rather go [and] buy it or they [planned] to come back whenever”*. (PN, 11 years)

#### Limited counseling time

A common challenge was insufficient time to provide women with counseling, particularly at facilities that provided contraceptive services to large numbers of employed women. Working women usually attended clinics during lunch breaks or before work. Consequently, many providers had little time for counseling despite clinics using an appointment-based system which facilitated spacing consultations:

*They get here, sister I did not come for this I literally have to be back at in, like 15 min … they come in their lunch time. So, when you want to sit with them and they … already made up their mind – so you just give them what they came for and then they leave ... give them condoms, try and give the message ... it is difficult*. (PN, 4 years)

Furthermore, at some facilities, there was pressure to work more quickly due to staff being “*one-stop shops*” and the provision of a broad range of health services. Providers remarked on the pressure and feasibility of providing services to a targeted number of patients. They believed this would limit their ability to provide comprehensive contraceptive counseling

*Exactly and also even with our patients … if I had to say … am I spending the adequate amount of time on counseling of, or helping patients or client – no. Because as, as much as you want to, like you want to help, like that patient can only take that much at that time* (PN, 4 years)

## Discussion

This study provides key insights into primary care providers' approaches to contraception and their experiences when delivering contraceptive services in Cape Town, SA. Our data suggests that providers' knowledge and attitudes influence the delivery of contraceptive counseling and prescribing practices, with no differences between facilities. Furthermore, despite providers across all five facilities displaying positive attitudes that prioritized women's contraceptive needs and desires, this study suggests that underlying factors, such as health system challenges, provider knowledge and perceptions as well as and women's bleeding preferences, influence how providers are delivering this service.

Overall, in-service SRH training was important, ensuring healthcare providers felt confident to provide effective contraceptive services. Inadequate knowledge and training affected providers' confidence when prescribing contraceptives and was a barrier to providing effective services (27, 30–33). In a recent South African study, providers reported that nurses required more specialized family training to address gaps in providing accurate information to women during contraceptive counseling (34). Studies across Africa have highlighted that in-service training could assist in mitigating provider bias (22, 35–37). Additionally, as was found elsewhere (28, 38), clinical training promoted trainees' provision of IUDs. Thus, despite local policy promoting the availability and accessibility of the IUD, without training in the promotion and insertion of the IUD, this might not be unachievable. However, other studies found differing results with regards to the impact of training; training had no effect (39), or it had a limited impact on some providers recommendations (35). It has been argued that training focusing exclusively on contraceptive safety is insufficient to mitigate against providers imposing restrictions on specific contraceptive methods (20). A key lesson that emerged during the introduction of the contraceptive implant in SA was the importance of providing high quality training (40). In this study, providers praised the in-service SRH training due to its depth and use of real-world scenarios in comparison to prior training they received. It also facilitated providers to reflect critically during assessments to determine the appropriateness of a specific contraceptive method which is both suitable and preferable for women. Consequently, the role of SRH training and how it impacts on providers prescribing practices requires further attention.

Providers also used clinical guidelines to assist with their clinical judgement. They considered multiple factors incorporated into decision trees to assess if women were medically suitable for their preferred contraceptive method. However, as was found in this study, providers may limit women's contraceptive choices regardless of available clinical and national guidelines (20). Furthermore, guidelines that promote a one-size-fits-all approach to contraceptive counseling may limit providers' autonomy and overlook the individual needs of women (41–43). Providers with negative attitudes toward algorithmic guidelines may rely on personal judgment, which could restrict women's access to a suitable contraceptive method, a common finding in studies related to the IUD (28). This study highlights the importance of accurate guidelines being clearly communicated to providers, particularly where contraindications to specific methods are present (40). These must speak to local experience. For example, as found elsewhere in SA (21), providers knew that the implant reduced contraceptive efficacy amongst HIV-infected women also using efavirenz. However, changing guidelines—to add condoms for these women (44)—was not seen as viable and created confusion amongst staff, resulting in them promoting implant removal and alternative methods. Providers did not report having avenues to address the prescribing dilemmas they experienced. These included issues such as the implants for HIV-infected women, and how to manage older women who insist on receiving injectables. Routine meetings usually discuss operational issues, staff and facility performance and targets. Forums and in-service training for staff to address common clinical dilemmas faced could both improve provider confidence, the health education they provide and ensure that women receive appropriate contraceptives of their choice.

Central to their professional identity, providers saw themselves as enablers of women's reproductive health decision-making by providing accurate information to guide their decisions, including method side effects. However, other SA studies conducted among women contraceptive users, found they often receive little to no information on side effects (17, 18). This mismatch could be explained by the constraints experienced by providers. They highlighted that they frequently experienced stock-outs which limited available methods. In addition, they were often unable to provide in-depth contraceptive counseling due to time constraints, imposed by both women and their own workloads. Irrespective, providing accurate information is essential to increase women's involvement in contraceptive decision-making and to promote the correct and consistent use of their preferred contraceptive method (45). In the United States, emerging research on contraception has focused on shared decision-making (46–48). This involves the provider facilitating the process toward agreement on an appropriate contraceptive method –providing accurate information, addressing misconceptions and helping women identify the suitability of the methods available, consistent with their contraceptive preferences (46).

In our study, providers engaged with women about their bleeding preferences when selecting a contraceptive method. This accords with findings from multi-country studies in Europe, North America and Latin America, that suggested bleeding preferences may influence contraception choices, where bleeding negatively impacted on women's daily activities (49, 50). Some women prefer a predictable bleeding whereas others prefer to reduce or eliminate bleeding and use hormonal contraception to achieve this (49, 50). Although information is very limited on women's bleeding pattern preferences in Africa (51), a scoping review highlighted that using contraception to achieve amenorrhea was more preferable in Europe, North America and Latin America compared to African countries. A few African studies indicate that some women perceive amenorrhea negatively and consider menstruation to be an indicator of fertility and wellbeing, which could influence contraceptive decision-making (51, 52). As was found in our study, bleeding preferences may differ across various populations due to individual, physical and social influences (51, 53), with some women wanting to avoid menstruation due to unaffordable sanitary products whereas younger women preferred a regular bleeding pattern to avoid parental suspicion of their use of contraception. Thus, providers in this study facilitated decision-making that aligned with women's bleeding preferences, which was influenced by their lived realities.

Providers displayed pre-existing attitudes that may have influenced contraceptive prescribing to younger and older women. As was found in other SA studies investigating the attitudes of providers toward providing contraceptive services to adolescent and young women (25, 26, 53, 54), our study findings highlight that when prescribing contraception to adolescents, providers continue to use stereotypes and value-based judgements, such as adolescents being irresponsible (25, 54, 55). Additionally, we found that providers had a negative attitude toward prescribing injectables to older women who request to use this method. Providers believed that injectable use might result in reduced bone density in older women and thus recommended women switch to a perceived safer method. The research predated the 2019 SA guidelines and contradicted the WHO guidelines which state that women over 40 can safely use injectables unless they have contraindications such as are smokers or are migraineurs (16, 56). This highlights the importance of timeous communication to providers about guideline changes, and discussion. While the clinical implications for the risk due to reduced bone density amongst older women using DMPA remains uncertain (29), this finding highlights that lack of accurate knowledge may bias providers against providing women their preferred contraceptive method.

As this study was confined to public primary health care facilities in urban Cape Town, SA, findings may not be generalizable to rural areas. However, facilities selected served a diverse population in terms of age, socio economic status and county of origin, and thus may be relevant for providers working in primary care facilities in the public sector elsewhere in SA. Although providers may have provided socially desirable responses, we aimed to mitigate this by using vignettes and follow-up questions, which explored providers' application of clinical judgment to motivate a specific contraceptive method and, the agreement with women's requests for a specific method.

The WHO emphasizes health care providers' critical role in ensuring women's right to accessing high quality contraceptive services (57). Achieving this, requires providers to be trained to prescribe and administer a range of contraceptive methods and deliver evidence-based information to ensure women make informed decisions about methods. Providers should enable women selecting a suitable method, without judgement, supporting their approval or refusal of suggested contraceptive methods. This study demonstrates that providers in a busy, pressurized work setting are committed to promoting contraceptive services that uphold women's preferences, despite their knowledge and attitudes influencing their counseling and prescribing practices. Ensuring that providers receive timeous, standardized evidence-based SRH training can support the provision of high-quality services, as prescribing practices varied and were based on the level of in-service training received. Outdated or inadequate knowledge influenced providers attitudes toward younger and older women and can be addressed by instituting avenues to resolve prescribing dilemmas. Additionally, shifting contraceptive counseling to focus on shared decision-making may promote providers' respect for women's autonomy during decision-making about method selection (48). However, this is an emergent area of research in family planning in other contexts such as the United States and requires further exploration within the SA and other LMIC contexts.

## Data availability statement

The datasets presented in this article are not readily available because informants were assured of confidentiality and anonymity, and consent was not obtained for sharing transcripts. Further enquiries can be directed to kulthum.fataar@uct.ac.za.

## Ethics statement

The studies involving human participants were reviewed and approved by University of Cape Town, Faculty of Health Sciences, Human Research Ethics Committee. The patients/participants provided their written informed consent to participate in this study.

## Author contributions

KF conceptualized the study, wrote the study proposal, conducted the fieldwork and data analysis, and drafted the article, as part of her work for a Master of Public Health. VZ fund raised for the study, reviewed the proposal, and contributed to article writing. JH gave input for and supervised the study proposal writing, fieldwork, and writing of the article. All authors contributed to the article and approved the submitted version.

## Funding

This study was funded by the Cape Higher Education Consortium (CHEC), in South Africa.

## Conflict of interest

The authors declare that the research was conducted in the absence of any commercial or financial relationships that could be construed as a potential conflict of interest.

## Publisher's note

All claims expressed in this article are solely those of the authors and do not necessarily represent those of their affiliated organizations, or those of the publisher, the editors and the reviewers. Any product that may be evaluated in this article, or claim that may be made by its manufacturer, is not guaranteed or endorsed by the publisher.

## References

[1] United Nations, *Department of Economic and Social Affairs, Population Division*. Contraceptive use by method 2019: Data booklet. Available online at: https://www.un.org/en/development/desa/population/publications/index.asp (accessed January 10, 2019).

[2] Bellizzi S Sobel HL Obara H Temmerman M. Underuse of modern methods of contraception: underlying causes and consequent undesired pregnancies in 35 low- and middle-income countries. Hum Reprod. (2015) 30:973–986. 10.1093/humrep/deu348 25650409

[3] Ewerling F Victora CG Raj A Coll CVN Hellwig F Barros AJD. Demand for family planning satisfied with modern methods among sexually active women in low- and middle-income countries: Who is lagging behind? Reprod Health. (2018) 15:1–10. 10.1186/s12978-018-0483-x 29510682 PMC5840731

[4] Ganatra B Faundes A. Role of birth spacing, family planning services, safe abortion services and post-abortion care in reducing maternal mortality. Best Pract Res Clin Obstet Gynaeco. (2016) 36:145–55. 10.1016/j.bpobgyn.2016.07.008 27640082

[5] Gerdts C Raifman S Daskilewicz K Momberg M Roberts S Harries J. Women's experiences seeking informal sector abortion services in Cape Town, South Africa: A descriptive study. BMC Womens Health. (2017) 17:1–10. 10.1186/s12905-017-0443-6 28969631 PMC5625615

[6] South African National Department of Health, Statistics South Africa, South African Medical Research Council, ICF. South Africa demographic and health survey. (2016). Available online at: https://www.samrc.ac.za/reports/SADHS2016 (accessed January 14, 2020).

[7] Lince-Deroche N Pleaner M Morroni C Mullick S Firnhaber C Harries J. Achieving universal access to sexual and reproductive health services : the potential and pitfalls for contraceptive services in South Africa. S Afr Health Rev. (2016) 1:95–108. 10.10520/EJC189315

[8] South African National Department of Health. National contraception clinical guidelines. Available online at: https://www.knowledgehub.org.za/elibrary/national-contraception-clinical-guidelines-2019 (accessed March 07, 2022).

[9] Ralph LJ Mccoy SI. Shiu K, Padian NS. Hormonal contraceptive use and women's risk of HIV acquisition: A meta-Analysis of observational studies. Lancet Infect Dis. (2015) 15:181–9. 10.1016/S1473-3099(14)71052-7 25578825 PMC4526270

[10] Colvin CJ Harrison A. Broadening the debate over HIV and hormonal contraception. Lancet Infect D. (2015) 15:135–136. 10.1016/S1473-3099(14)71076-X 25578824 PMC4580273

[11] Ahmed K Baeten JM Beksinska M Bekker LG Bukusi EA Donnell D. HIV incidence among women using intramuscular depot medroxyprogesterone acetate, a copper intrauterine device, or a levonorgestrel implant for contraception: A randomised, multicentre, open-label trial. Lancet. (2019) 394:303–13. 10.1016/S0140-6736(19)31288-7 31204114 PMC6675739

[12] World Health Organization. Contraceptive eligibility for women at high risk of HIV. Guidance statement: Recommendations on contraceptive methods used by women at high risk of HIV. Available online at: https://www.who.int/reproductivehealth/publications/contraceptive-eligibility-women-at-high-risk-of-HIV/en/ (accessed March 08, 2020). 31503432

[13] Chersich MF Wabiri N Risher K Shisana O Celentano D Rehle T. Contraception coverage and methods used among women in South Africa: A national household survey. S Afr Med J. (2017) 107:307–314. 10.7196/SAMJ.2017.v107i4.12141 28395681

[14] Seutlwadi L Peltzer K Mchunu G Tushana BO. Contraceptive use and associated factors among South African youth (18–24 years): A population-based survey. S Afr J Obstet Gynaecol. (2012) 8:43–47. Available online at: https://www.ajol.info/index.php/sajog/article/view/76924 (accessed January 14, 2020).

[15] Adeagbo OA Mullick S Pillay D Chersich MF Morroni C Naidoo N. Uptake and early removals of Implanon NXT in South Africa: Perceptions and attitudes of healthcare workers. S Afr Med J. (2017) 107:822–826. 10.7196/SAMJ.2017.v107i10.12821 29397681

[16] World Health Organisation. Medical eligibility criteria wheel for contraceptive use. Geneva: World Health Organisation (2015).

[17] Harries J Constant D Wright V Morroni C Müller A Colvin CJ. multidimensional approach to inform family planning needs, preferences and behaviours amongst women in South Africa through body mapping. Reprod Health. (2019) 16:1–11. 10.1186/s12978-019-0830-6 31694648 PMC6836666

[18] Marlow HM Maman S Moodley D Curtis S. Postpartum family planning service provision in Durban, South Africa: client and provider perspectives. Health Care Women Int. (2014) 35:175–99. 10.1080/07399332.2013.815753 23998760 PMC4083758

[19] de Irala J Osorio A Carlos S Lopez-del Burgo C. Choice of birth control methods among European women and the role of partners and providers. Contraception. (2011) 84:558–564. 10.1016/j.contraception.2011.04.004 22078183

[20] Solo J Festin M. Provider bias in family planning services: A review of its meaning and manifestations. Glob Health Sci Pract. (2019) 7:371–385. 10.9745/GHSP-D-19-00130 31515240 PMC6816811

[21] Lince-Deroche N Hendrickson C Moolla A Kgowedi S Mulongo M. Provider perspectives on contraceptive service delivery: findings from a qualitative study in Johannesburg, South Africa. BMC Health Serv Res. (2020) 20:1–10. 10.1186/s12913-020-4900-9 32085756 PMC7035764

[22] Sieverding M Schatzkin E Shen J Liu J. Bias in contraceptive provision to young women among private health care providers in South West Nigeria. Int Perspect Sex Reprod Health. (2018) 44:19–29. 10.1363/44e5418 30028307

[23] Green J Thorogood N. Qualitative Methods for Health Research. 3rd ed London: Sage Publications (2014).

[24] Starling S Burgess S Bennette N Neighbor H. Literature review and expert interviews on provider bias in the provision of youth contraceptive services: research summary and synthesis. (2017). Available online at: https://www.pathfinder.org/wp-content/uploads/2018/10/BB_Research-Synthesis.pdf (accessed June 9, 2022).

[25] Müller A Röhrs S Hoffman-Wanderer Y Moult K. “You have to make a judgment call”—Morals, judgments and the provision of quality sexual and reproductive health services for adolescents in South Africa. Soc Sci Med. (2016) 148:71–78. 10.1016/j.socscimed.2015.11.048 26688551

[26] Wood K Jewkes R. Blood blockages and scolding nurses: barriers to adolescent contraceptive use in South Africa. Reprod Health Matters. (2006) 14:109–118. 10.1016/S0968-8080(06)27231-8 16713885

[27] Gutin SA Mlobeli R Moss M Buga G Morroni C. Survey of knowledge, attitudes and practices surrounding the intrauterine device in South Africa. Contraception. (2011) 83:145–150. 10.1016/j.contraception.2010.07.009 21237340

[28] Daniele MAS Cleland J Benova L Ali M. Provider and lay perspectives on intra-uterine contraception: A global review. Reprod Health. (2017) 14:1–11. 10.1186/s12978-017-0380-8 28950913 PMC5615438

[29] Downs R W Jr Yazbeck CF. Osteoporosis after long-term Depot Medroxyprogesterone. We need more data on fracture risk. J Women's Health. (2015) 24:619–20. 10.1089/jwh.2015.29000-rwd 26263280

[30] Politi MC Estlund A Milne A Buckel CM Peipert JF Madden T. Barriers and facilitators to implementing a patient-centred model of contraceptive provision in community health centers. Contracept Reprod Med. (2016) 1:1–9. 10.1186/s40834-016-0032-3 29201410 PMC5693580

[31] Dehlendorf C Levy K Ruskin R Steinauer J. Health care providers' knowledge about contraceptive evidence: A barrier to quality family planning care? Contraception. (2010) 81:292–298. 10.1016/j.contraception.2009.11.006 20227544 PMC2892417

[32] Silumbwe A Nkole T Munakampe MN Milford C Cordero JP Kriel Y. Community and health systems barriers and enablers to family planning and contraceptive services provision and use in Kabwe District, Zambia. BMC Health Serv Res. (2018) 18:1–11. 10.1186/s12913-018-3136-4 29855292 PMC5984360

[33] Akers AY Gold MA Borrero S Santucci A Schwarz EB. Providers' perspectives on challenges to contraceptive counseling in primary care settings. J Womens Health. (2010) 19:1163–70. 10.1089/jwh.2009.1735 20420508 PMC2940510

[34] Kriel Y Milford C Cordero JP Suleman F Steyn PS Smit JA. Quality of care in public sector family planning services in KwaZulu-Natal, South Africa: A qualitative evaluation from community and health care provider perspectives. BMC Health Serv Res. (2021) 2021:1246. 10.1186/s12913-021-07247-w 34789232 PMC8600736

[35] Schwandt HM Speizer IS Corroon M. Contraceptive service provider imposed restrictions to contraceptive access in urban Nigeria. BMC Health Serv Res. (2017) 17:1–9. 10.1186/s12913-017-2233-0 28403858 PMC5389090

[36] Tumlinson K Okigbo CC Speizer IS. Provider barriers to family planning access in urban Kenya. Contraception. (2015) 92:143–151. 10.1016/j.contraception.2015.04.002 25869629 PMC4506861

[37] Godia PM Olenja JM Lavussa JA Quinney D Hofman JJ van den Broek N. Sexual reproductive health service provision to young people in Kenya; health service providers' experiences. BMC Health Serv Res. (2013) 13:476. 10.1186/1472-6963-13-476 24229365 PMC4225671

[38] Thompson KMJ Rocca CH Stern L Morfesis J Goodman S Steinauer J. Training contraceptive providers to offer intrauterine devices and implants in contraceptive care: A cluster randomized trial. Am J Obstet Gynecol. (2018) 218:597.e1–597.e7. 10.1016/j.ajog.2018.03.016 29577915 PMC5970088

[39] Speizer IS Hotchkiss DR Magnani RJ Hubbard B Nelson K. Do service providers in Tanzania unnecessarily restrict clients' access to contraceptive methods? Int Fam Plan Perspect. (2000) 26:13–42. 10.2307/2648285

[40] Pleaner M Morroni C Smit J Lince-Deroche N Chersich M Mullick S. Lessons learnt from the introduction of the contraceptive implant in South Africa. S Afr Med J. (2017) 107:933–938. 10.7196/SAMJ.2017.v107i11.12805 29399422

[41] Jansson M Ala-Kokko T Ylipalosaari P Syrjälä H Kyngäs H. Critical care nurses' knowledge of, adherence to and barriers towards evidence-based guidelines for the prevention of ventilator-associated pneumonia—A survey study. Intensive Crit Care Nurs. (2013) 29:216–227. 10.1016/j.iccn.2013.02.006 23566622

[42] van de Steeg L Langelaan M Ijkema R Nugus P Wagner C. Improving delirium care for hospitalized older patients. A qualitative study identifying barriers to guideline adherence. J Eval Clin Pract. (2014) 20:813–819. 10.1111/jep.12229 25081423

[43] Abrahamson KA Fox RL Doebbeling BN. Facilitators and barriers to clinical practice guideline use among nurses. Am J Nurs. (2012) 112:26–36. 10.1097/01.NAJ.0000415957.46932.bf 22705494

[44] South African National Department of Health. Technical brief: drug interactions with progestin subdermal implants. 2014. Available online at: http://www.health.gov.za/index.php/circulars/category/551-2014-circular (accessed March 08, 2020).

[45] Abdel-Tawab N RamaRao S. Do improvements in client-provider interaction increase contraceptive continuation? Unraveling the puzzle. Patient Educ Couns. (2010) 81:381–7. 10.1016/j.pec.2010.10.010 21074962

[46] Chen M Lindley A Kimport K Dehlendorf C. An in-depth analysis of the use of shared decision making in contraceptive counseling. Contraception. (2019) 99:187–91. 10.1016/j.contraception.2018.11.009 30471263

[47] Dehlendorf C Grumbach K Schmittdiel JA Steinauer J. Shared decision making in contraceptive counseling. Contraception. (2017) 95:452–455. 10.1016/j.contraception.2016.12.010 28069491 PMC5466847

[48] Dehlendorf C Krajewski C Borrero S. Contraceptive counseling: best practices to ensure quality communication and enable effective contraceptive use. Clin Obstet Gynecol. (2014) 57:659–673. 10.1097/GRF.0000000000000059 25264697 PMC4216627

[49] Szarewski A von Stenglin A Rybowski S. Women's attitudes towards monthly bleeding: Results of a global population-based survey. Eur J Contracept Reprod Health Care. (2012) 17:270–283. 10.3109/13625187.2012.684811 22758651

[50] Nappi RE Fiala C Chabbert-Buffet N Häusler G Jamin C Lete I. Women's preferences for menstrual bleeding frequency: results of the Inconvenience Due to Women's Monthly Bleeding (ISY) survey. Eur J Contracept Reprod Health Care. (2016) 21:242–250. 10.3109/13625187.2016.1154144 27010535

[51] Polis CB Hussain R Berry A. There might be blood: A scoping review on women's responses to contraceptive-induced menstrual bleeding changes. Reprod Health. (2018) 15:114. 10.1186/s12978-018-0561-0 29940996 PMC6020216

[52] Laher F Todd CS Stibich MA Phofa R Behane X Mohapi L. Role of menstruation in contraceptive choice among HIV-infected women in Soweto, South Africa. Contraception. (2010) 81:547–51. 10.1016/j.contraception.2009.12.010 20472125

[53] Lince-Deroche N Hargey A Holt K Shochet T. Accessing sexual and reproductive health information and services: A mixed methods study of young women's needs and experiences in Soweto, South Africa. Afr J Reprod Health. (2015) 19:73–81. 10.10520/EJC168621 26103697

[54] Holt K Lince-Deroche N Hargey A Struthers H Nkala B McIntyre J. Assessment of service availability and health care workers' opinions about young women's sexual and reproductive health in Soweto, South Africa. Afr J Reprod Health. (2012) 16:283–293. 10.10520/EJC121528 22916560

[55] Jonas K Roman N Reddy P Krumeich A van den Borne B Crutzen R. Nurses' perceptions of adolescents accessing and utilizing sexual and reproductive healthcare services in Cape Town, South Africa: A qualitative study. Int J Nurs Stud. (2019) 97:84–93. 10.1016/j.ijnurstu.2019.05.008 31200221

[56] South African National Department of Health. National contraception guidelines: A companion to national contraception and fertility planning policy and service delivery guidelines. (2012). Available online at: https://www.gov.za/documents/national-contraception-clinical-guidelines (accessed March 08, 2020).

[57] World Health Organization. Ensuring human rights in the provision of contraceptive information and services. (2014). Available online at: https://www.who.int/reproductivehealth/publications/family_planning/human-rights-contraception/en/(accessed March 19, 2020). 24696891

[58] Fataar K. An exploration of the knowledge, attitudes and practices of primary health care providers providing contraceptive and family planning services in Cape Town, South Africa: A qualitative study. [MPH, thesis]. University Cape Town. 2020. Available online at: https://open.uct.ac.za/handle/11427/32672 (accessed March 25, 2022).

