# Supplementary material for: Providers' approaches to contraceptive provision in Cape Town
Source: Front Glob Womens Health. 2022 Sep 14;3:917881. doi: 10.3389/fgwh.2022.917881 (PMC9515548; doi:10.3389/fgwh.2022.917881)
Supplement: Supplementary file 1 [file Table_1.DOCX]

**Interview Schedule**

**General:**

Introductory comments about the researcher and the study. Following this, the informed consent form will be explained. Remind participant that we can have a 10 minute break after 45 minutes (or sooner if needed)

Introductions:

- What qualifications do you have,
- how long have you been a nurse,
- how long have you been providing family planning services and where?
- How do you feel about contraceptive counselling and prescribing?
- What are the contraceptives prescribed here?

Knowledge:

- What policies and clinical guidelines inform your practice?
- Do you receive training on contraceptives, prescribing and counselling? When/how often? How do you keep yourself up to date?
- Does the knowledge and training you have received feel adequate for various counselling/prescribing situations (do you feel more)? Does it allow you to feel more confident in your practice and making clinical decisions at the clinic? If not, why?
- What do you think are the most popular contraceptive methods prescribed? Why?
- What do you think are the most popular contraceptive methods requested by patients? Why?
- Tell me about the things (factors) you think about (knowledge and skills you have) when thinking about what is best to prescribe/not prescribe a contraceptive method to a patient? When would you not prescribe certain contraceptives?
- Why do you think a patient might discontinue contraception use?
- What do you think is important for patients to know (information you provide) about contraception?

**Present vignettes and discuss:**

- How would you counsel this person? Why would you choose one method for this case over another?
- How would you feel about providing contraception to this person?

Practices:

- Tell me about a typical session providing family planning services? What happens/doesn’t happen? What is your role? What is the patient’s role? What informs your decisions?
- What professional challenges do you experience when providing contraceptive counselling? When prescribing? (link here to knowledge – are they always able to provide service according to training and knowledge received? What situations are they unable to manage)
- What happens when a patient requests a particular contraceptive method? How often do patients change their mind after counselling?

Attitudes:

- Who do you think is best suited for 1) the pill, 2) IUD 3) the injection 4) Implanon 5) IUD. Why?
- Do you ever disagree with patients request for a particular contraceptive method? What influences you to disagree? How do you go about dealing with this?
- How confident do you feel providing different contraceptive methods? What makes you feel confident about this? Do you feel more confident prescribing some than others? How confident do you feel about prescribing newer contraceptives?
- Would you provide a contraceptive method you felt uncertain about? How would you go about addressing this?
- Tell me about any experiences where your personal beliefs impacted on what you chose to prescribe or to counsel on?
- How do you feel about your role as someone prescribing and counselling contraceptives? Do you feel you have significant influence on patient decision making? How do you feel about shared decision-making? (Do you see yourself and patient as equal in decision making)
- How do you feel about patients who show low adherence or frequently discontinue contraceptives? How do you manage such situations?

Recommendations**:**

- What recommendations do you have to improve family planning services?

**Vignette 1:**

A 17-year-old patient attends the clinic, asking to use the oral contraceptive pill. She lives quite far from the clinic, so travelling is not easy for her. She lives with her single mother and 3 younger siblings in a 2 bedroom home. Her mother is not aware she is seeking contraceptives. She is currently not in a steady relationship, however she has been sexually active for a few months.

**Vignette 2:**

A 25-year-old patient attends the clinic; she has a 5 year old child from a previous relationship and does not want to fall pregnant any time soon as she intends to study a 3 year degree which she will start next year. She previously used the injection but hated the weight gain and side-effects so she failed to attend her follow up sessions. She is currently working part-time and living with her parents. She has read about the IUD and is wondering if that might be a good option.
