# Supplementary material for: Providers' approaches to contraceptive provision in Cape Town
Source: Front Glob Womens Health. 2022 Sep 14;3:917881. doi: 10.3389/fgwh.2022.917881 (PMC9515548; doi:10.3389/fgwh.2022.917881)
Supplement: Supplementary file 2 [file Table_2.DOCX]

Vignette 1:

A 17 year old patient attends the clinic, asking to use the oral contraceptive pill. She lives quite far from the clinic, so travelling is not easy for her. She lives with her single mother and 3 younger siblings in a 2 bedroom home. Her mother is not aware she is seeking contraceptives. She is currently not in a steady relationship, however she has been sexually active for a few months.

Vignette 2:

A 25 year old patient attends the clinic; she has a 5 year old child from a previous relationship and does not want to fall pregnant any time soon as she intends to study a 3 year degree which she will start next year. She previously used the injection, but hated the weight gain and side-effects so she failed to attend her follow up sessions. She is currently working part-time and living with her parents. She has read about the IUD and is wondering if that might be a good option.
